# Evaluation of Anthocyanin Content, Antioxidant Potential and Antimicrobial Activity of Black, Purple and Blue Colored Wheat Flour and Wheat-Grass Juice against Common Human Pathogens

**DOI:** 10.3390/molecules25245785

**Published:** 2020-12-08

**Authors:** Natasha Sharma, Vandita Tiwari, Shreya Vats, Anita Kumari, Venkatesh Chunduri, Satveer Kaur, Payal Kapoor, Monika Garg

**Affiliations:** 1 Agri-Biotechnology Division, National Agri-Food Biotechnology Institute, Mohali 140306, India; natasha.micro@gmail.com (N.S.); vanditatiwari2017@gmail.com (V.T.); anitaani91@gmail.com (A.K.); chundurivenkatesh@gmail.com (V.C.); satveerkaur5555@gmail.com (S.K.); ssspayalkapur@gmail.com (P.K.); 2 Department of Biochemistry, Panjab University, Chandigarh 160014, India; shreyavats796@gmail.com

**Keywords:** black wheat, purple wheat, black wheat-grass, antioxidant, minimum inhibitory concentration (MIC), minimum microbicidal concentration (MMC)

## Abstract

The present study aimed to analyze the antioxidant and antimicrobial activity of anthocyanins extracted from colored wheat flour and wheat-grass juice against human pathogens. The total anthocyanin content and antioxidant potential in colored wheat flour and wheat-grass juice extracts were significantly higher than white flour and wheat-grass juice extracts. Ultra-performance liquid chromatography showed the maximum number of anthocyanin peaks in black wheat, with delphinidin-3-o-galactoside chloride, delphinidin-3-o-glucoside chloride, and cyanindin-3-o-glucoside chloride as the major contributors. Among flour extracts, maximum zones of inhibition against *Staphylococcus aureus* (MTCC 1934), *Pseudomonas aeruginosa* (MTCC 1434), *Escherichia coli*, and *Candida albicans* (MTCC 227) were produced by black flour extract, having the highest anthocyanin content. It exhibited a minimum microbicidal concentration (MMC) of 200 mg/mL against *E. coli* and *C. albicans*; and 100 and 150 mg/mL against *S. aureus* and *P. aeruginosa*, respectively. Black and purple flour extracts exhibited a minimum inhibitory concentration (MIC) of 50 mg/mL against *S. aureus* and *P. aeruginosa*. White flour extracts did not show MMC against *E. coli* and *C. albicans.* Among wheat-grass juice extracts, black wheat-grass was most effective and showed an MIC of 100–150 mg/mL against all pathogens. It exhibited an MMC of 200 mg/mL against *S. aureus* and *P. aeruginosa*. Hence, anthocyanin-rich colored wheat could be of nutraceutical importance.

## 1. Introduction

Staple foods constitute the majority of a particular diet and supply most of the total energy needs and nutrient requirements of populations. Wheat is one of the most important food crops in the world and is the main source of energy in developing nations [1]. It provides substantial amounts of many beneficial nutrients like proteins; vitamin B and E; dietary fibers; minerals like iron and zinc; and phytochemicals such as phenolics and flavonoids [2]. Wheat-grass, the young 10 days old shoots of wheat, *Triticum aestivum*, is the storehouse of several nutrients that are important for maintaining good health [3]. It is a rich source of proteins; essential amino acids; carbohydrates; dietary fibers; chlorophyll; vitamin A, B, C, and E; minerals such as iron, calcium, magnesium, and selenium [4,5]; and some phenolic compounds including flavonoids [6]. Phenolic and flavonoid compounds decrease cell damage caused by oxidative stress because these are capable of scavenging free radicals and possess ferric reducing abilities [7,8]. Hence, wheat-grass juice is known as a superfood because of its benefits for human health [9].

Wheat and wheat-grass are reported to possess antimicrobial activity against many common human pathogens [10,11]. Choi et al. [12] found wheat seed ethyl acetate extracts of different wheat varieties to be effective against the pathogens *Escherichia coli*, *Salmonella typhimurium*, and *Staphylococcus aureus* using well diffusion assays. Kim et al. [13] studied the antimicrobial effect of wheat germ against *S. aureus*, *E. coli*, *S. typhimurium*, and *Bacillus cereus* and found the potential of wheat germ as a natural antimicrobial and food preservative agent. Wheat-grass extracts are also reported to show antibacterial activity against many bacteria like *Yersinia enterocolitica* and *Listeria monocytogenes*, which are some foodborne pathogens [14,15]. Ashok [16] also reported the antibacterial activity of wheat-grass juice against the pathogens *E. coli*, *Pseudomonas aeruginosa*, *S. aureus*, and *Candida albicans*.

**Figure 1:**
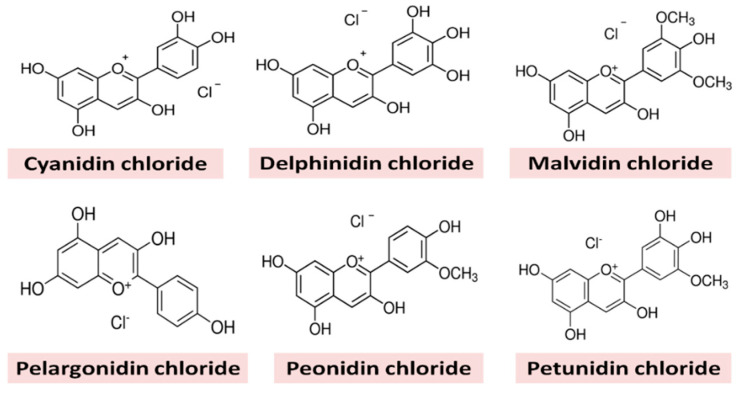
Structures of different anthocyanins present in wheat.

Commonly cultivated wheat (*T. aestivum*) is amber in color and has a much fewer amount of anthocyanins, but colored wheat varieties (blue, black, purple, and red) of *T. aestivum* are rich in anthocyanins and other phytochemicals and are becoming popular around the world nowadays [17,18]. Anthocyanin is considered as one of the flavonoids, although it has a positive charge at the oxygen atom of the C-ring of basic flavonoid structure. It is also called the flavylium (2-phenylchromenylium) ion. Anthocyanins are present in glycosylated forms of six anthocyanidins, namely, cyanidin, delphinidin, pelargonidin, peonidin, petunidin, and malvidin (Figure 1). Colored wheat contains a high amount of anthocyanins, which are bioactive compounds with numerous health benefits, including preventing and fighting various chronic diseases such as cancer, cardiovascular diseases, diabetes, inflammation, obesity, aging, liver dysfunction, and hypertension [17,19,20]. Besides this, anthocyanins are also reported to possess a strong antimicrobial activity against different Gram-positive and Gram-negative human pathogens [21,22]. Lacombe et al. [23] demonstrated that anthocyanin-rich American cranberry showed a reduction in the growth of *E. coli* after treatment with anthocyanin extracts. Red cabbage, sour cherry pomace, and *Lonicera caerulea* L. (haskap) berries are good sources of anthocyanins and their extracts are used as natural antimicrobial agents to prevent foodborne outbreaks related to *E. coli*, *S. aureus*, *L. monocytogenes*, *S. typhimurium*, and *B. cereus* [24,25].

Although an extensive body of scientific literature exists on the antimicrobial effects of anthocyanins extracted from fruits and vegetables, little is known about their antimicrobial properties in grains [26]. In addition, plant-based natural antimicrobial or biocontrol agents with therapeutic properties have attracted attention in recent years in the control of human and plant infectious diseases. The National Agri-Food Biotechnology Institute (NABI), Punjab, India has developed colored wheat varieties (black, purple, and blue) of *T. aestivum* rich in anthocyanins and the present research work was designed with the objective to elucidate the antagonistic activity of anthocyanins extracted from black-, purple-, and blue-colored wheat flour and wheat-grass against some common human pathogens viz., *S. aureus*, *P. aeruginosa*, *E. coli*, and *C. albicans*.

## 2. Results

### 2.1. Total Anthocyanin Content in Wheat Flour and Wheat-Grass Juice

**Figure 2:**
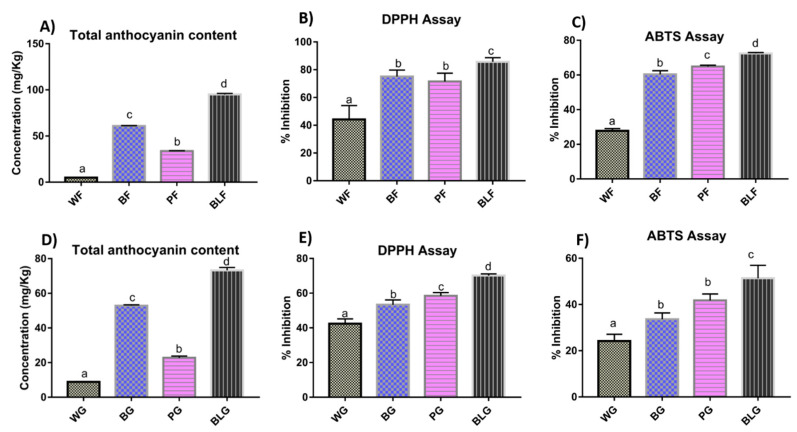
Total anthocyanin content (**A**,**D**); and antioxidant potential of wheat flour and wheat-grass juice using DPPH (2,2-diphenyl-1-picrylhydrazyl) (**B**,**E**) and ABTS ((2,2′-azino-bis(3-ethylbenzothiazoline-6-sulfonic acid) (**C**,**F**) assays. Each bar represents the mean of three replicates ± standard deviation. Significant differences in the bar heights analyzed according to one-way ANOVA. Different letters above bars in each graph represent significantly different values (*p* < 0.05) designated with a < b < c. Similar letters above the bars represent values being statistically on par. WF—white wheat flour; BF—blue wheat flour; PF—purple wheat flour; BLF—black wheat flour; WG—white wheat-grass; BG—blue wheat-grass; PG—purple wheat-grass; BLG—black wheat-grass.

The TAC of wheat flour ranged between 6.61 and 95.04 mg/kg and followed the order black > blue > purple > white. All colored wheat varieties had significantly higher TAC content than white wheat (Figure 2a). In the case of wheat-grass juice, TAC content ranged between 8.88 and 72.26 mg/kg. The highest TAC content was observed in black wheat-grass juice, followed by blue and purple wheat-grass juice and the lowest was in white wheat-grass juice (Figure 2d).

### 2.2. Antioxidant Potential of Wheat Flour and Wheat-Grass Juice

#### 2.2.1. DPPH Assay

The percent inhibition of DPPH (2,2-diphenyl-1-picrylhydrazyl) by different colored wheat flours ranged between 44.20 and 85.69%, with maximum inhibition obtained in the case of black wheat and minimum in white wheat. Purple and blue wheat flours had statistically similar DPPH inhibitory activity. The antioxidant potential of colored wheat flours was significantly higher than white wheat flour (Figure 2b). In the case of wheat-grass juices, percent inhibition of DPPH ranged between 42.46 and 70.19%. Among colored wheat-grass juices, maximum inhibition was shown by black wheat-grass juice followed by purple and blue wheat-grass juice, whereas the lowest activity was shown by white wheat-grass juice (Figure 2e).

#### 2.2.2. ABTS Assay

The percent inhibition of ABTS (2,2′-azino-bis(3-ethylbenzothiazoline-6-sulfonic acid) by different wheat flours ranged between 27.78 and 71.11% and followed the order black > purple > blue > white (Figure 2c). In the case of wheat-grass juices, the percent inhibition ranged between 24.18 and 51.40%. The highest antioxidant activity was shown by black wheat-grass juice followed by purple and blue wheat-grass juice, whose antioxidant activities were statistically on par with each other. The lowest activity was observed in the case of white wheat-grass juice (Figure 2f).

### 2.3. Determination of Anthocyanins by UPLC

**Figure 3:**
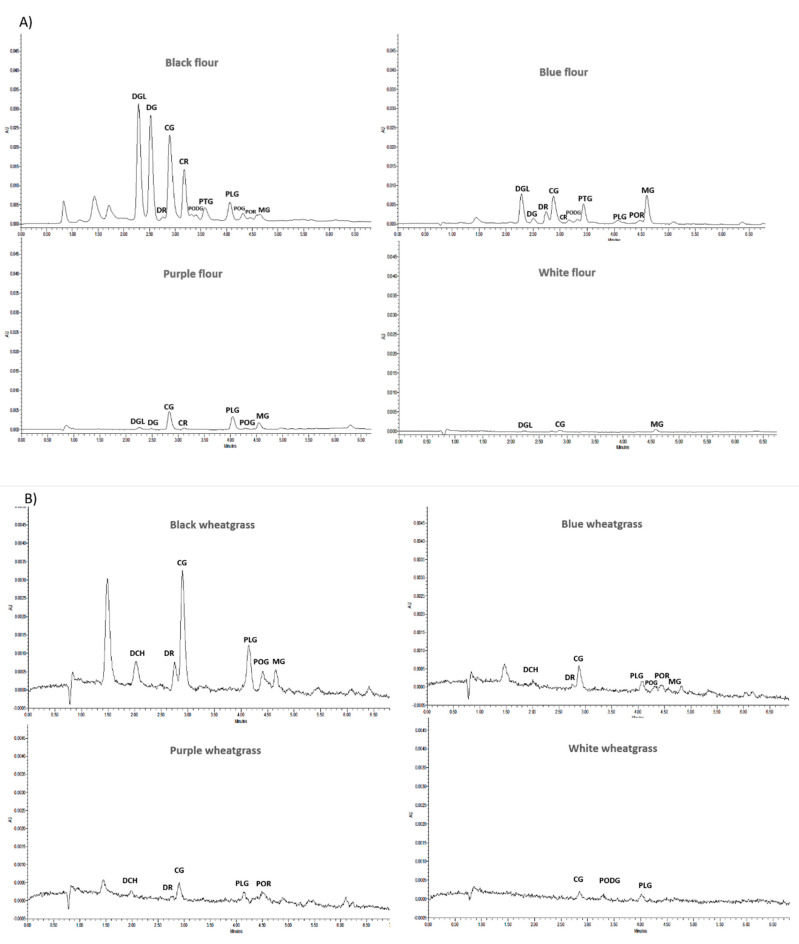
Chromatographs of anthocyanins present in different colored (**A**) wheat flours and (**B**) wheat-grass. DGl—delphinidin-3-o-galactoside chloride; DG—delphinidin-3-o-glucoside chloride; DR—delphinidin-3-o-rutinoside chloride; CG—cyanindin-3-o-glucoside chloride; PODG—peonidin-3,5-di-o-glucoside chloride; PTG—petunidin-3-o-glucoside chloride; PLG—pelargonidin 3-o-glucoside chloride; POG—peonidin-3-o-glucoside chloride; POR—peonidin-3-o-rutinoside chloride; MG—malvidin-3-o-glucoside chloride; CR—cyanindin-3-o-rutinoside chloride; DCH—delphin chloride (delphinidin-3,5-di*-*o-glucoside chloride).

UPLC analysis showed characteristic peak patterns for black, purple, blue, and white wheat flour and wheat-grass juice anthocyanin extracts. In the case of wheat flours, the maximum number of anthocyanin peaks (11) was identified in black wheat extracts followed by blue (10) and purple wheat flour extracts (6). White wheat flour extracts showed only two small peaks depicting trace amounts of anthocyanins (Figure 3).

Among 11 anthocyanin peaks in black wheat flour anthocyanin extracts, the highest concentration was observed for delphinidin-3-o-galactoside chloride (29.14 ppm) followed by delphinidin-3-o-glucoside chloride (25.64 ppm), cyanindin-3-o-glucoside chloride (20.50 ppm), and cyanindin-3-o-rutinoside chloride (11.14 ppm). The other peaks were observed for delphinidin-3-o-rutinoside chloride, peonidin-3,5-di-o-glucoside chloride, petunidin-3-o-glucoside chloride, pelargonidin 3-o-glucoside chloride, peonidin-3-o-glucoside chloride, peonidin-3-o-rutinoside chloride, and malvidin-3-o-glucoside chloride (Table 1).

The blue wheat flour anthocyanin extract chromatograph consisted of 10 identified anthocyanin peaks similar to that of black wheat flour extracts, except it did not contain peonidin-3-o-glucoside chloride. In the case of blue wheat extracts, the highest concentration was observed in the case of malvidin-3-o-glucoside chloride (5.5 ppm), followed by delphinidin-3-o-galactoside chloride (4.95 ppm), cyanindin-3-o-glucoside chloride (4.5 ppm), and petunidin-3-o-glucoside chloride (3.18 ppm).

Purple wheat flour anthocyanin extract chromatographs showed the presence of six identified anthocyanin peaks, with the highest concentration observed for cyanindin-3-o-glucoside chloride (2.64 ppm) followed by pelargonidin 3-o-glucoside chloride (1.88 ppm) and malvidin-3-o-glucoside chloride (1.32 ppm). The other peaks were identified as delphinidin-3-o-galactoside chloride, delphinidin-3-o-glucoside chloride, and cyanindin-3-o-rutinoside chloride.

White wheat flour extract chromatographs showed the presence of trace amounts of three identified anthocyanins corresponding to delphinidin-3-o-galactoside chloride, cyanindin-3-o-Glucoside chloride, and malvidin-3-o-glucoside chloride.

Wheat-grass juice extracts had a lesser number of anthocyanin peaks in comparison to wheat flour extracts. Black wheat-grass juice anthocyanin extract chromatographs showed six identified peaks, with the highest concentration observed in the case of cyanindin-3-o-glucoside chloride (4.74 ppm) followed by pelargonidin 3-o-glucoside chloride (2.77 ppm). The other peaks observed were delphinidin-3-o-rutinoside chloride, peonidin-3-o-glucoside chloride, malvidin-3-o-glucoside chloride, and delphin chloride.

Purple wheat-grass juice anthocyanin extract chromatographs also showed the presence of six identified peaks and had similar pattern as that of black wheat-grass juice extracts, except for it consisted of peonidin-3-o-rutinoside chloride instead of malvidin-3-o-glucoside chloride. However, the concentration of all anthocyanins in purple wheat-grass extracts was less than 1 ppm, with the highest observed in the case of pelargonidin 3-o-glucoside chloride (0.41 ppm).

Delphinidin-3-o-rutinoside chloride (2.36 ppm) was found to be highest in blue wheat-grass juice anthocyanin extracts. The other four identified anthocyanin peaks viz., pelargonidin 3-o-glucoside chloride, peonidin-3-o-glucoside chloride, peonidin-3-o-rutinoside chloride, and malvidin-3-o-glucoside chloride were less than 1 ppm in concentration.

White wheat-grass juice anthocyanin extract chromatographs showed the presence of three peaks viz., cyanindin-3-o-glucoside chloride (0.37 ppm), peonidin-3,5-di-o-glucoside chloride (0.26 ppm), and pelargonidin 3-o-glucoside chloride (0.35 ppm).

### 2.4. Antimicrobial Activity of Colored Wheat Anthocyanins Against Microbial Strains Using Agar-Overlay Method

The results revealed high antimicrobial activity of anthocyanin extracts from colored wheat flour and wheat-grass juice against the human pathogens in comparison to white wheat. The antimicrobial activity of the extracts was dose-dependent.

**Figure 4:**
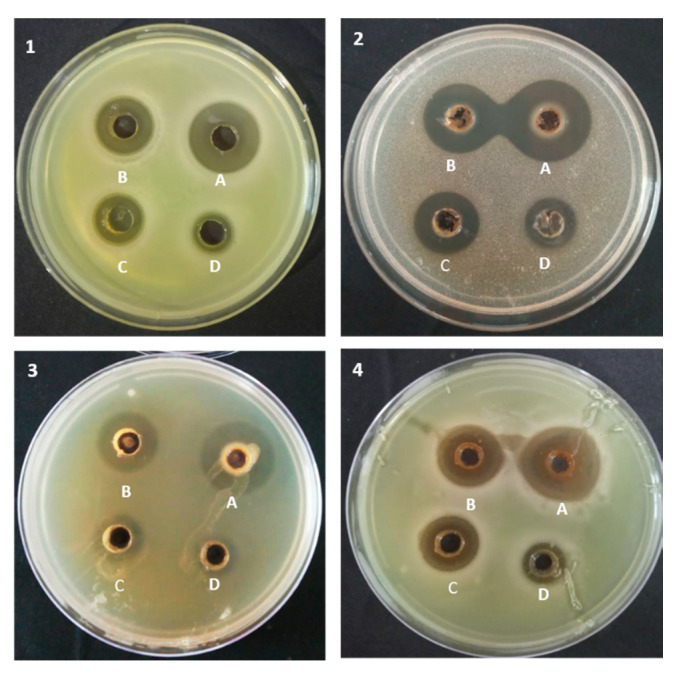
Antagonistic activity of anthocyanin extracts of black wheat flour against. (**1**) *Staphylococcus aureus*, (**2**) *Pseudomonas aeruginosa*, (**3**) *Escherichia coli*, and (**4**) *Candida albicans* at different anthocyanin extract concentrations; A = 200 mg/mL; B = 150 mg/mL; C = 100 mg/mL; D = 50 mg/mL.

Black wheat flour anthocyanin extracts (50–200 mg/mL) showed antimicrobial activity against all the pathogens. Among wheat flour anthocyanin extracts, antimicrobial activity followed the order black wheat > purple wheat > blue wheat > white wheat. All extracts produced clear halo zones against *S. aureus*, *P. aeruginosa*, and *C. albicans* (Figure 4). Black wheat flour extracts (200 mg/mL) showed significantly higher zones of inhibition against C. albicans (2.57 cm) and S. aureus (2.50 cm) amongst all flour extracts. Black and purple wheat flour extracts (200 mg/mL) showed maximum but statistically similar zones of inhibition against *P. aeruginosa* (2.73 and 2.60 cm) and *E. coli* (2.50 and 2.37 cm) in comparison to other flour extracts, as shown in Table 2.

At this concentration, blue wheat flour did not show any activity against *S. aureus* and *C. albicans*. Black and purple wheat flour extracts showed activity against all the pathogens, even at low extract concentration (50 mg/mL), while at this concentration, white flour extracts did not show any activity against *C. albicans*.

Black wheat-grass juice extracts (200 mg/mL) produced significantly higher zones of inhibition against *P. aeruginosa* (2.60 cm) and *C. albicans* (2.37 cm) in comparison to all other extracts. Black as well as purple wheat-grass juice extracts (200 mg/mL) showed significantly higher zones of inhibition against *S. aureus* (2.23 and 2.20 cm) and *E. coli* (1.93 and 2.0 cm). Black and purple wheat-grass juice extracts showed promising activity against all four pathogens, even up to 50 mg/mL extract concentration, while at this concentration, blue wheat flour did not show any activity against *S. aureus* and *C. albicans*. White wheat-grass juice extracts also did not show any activity against *C. albicans* at this concentration.

### 2.5. Minimum Inhibitory Concentration (MIC) and Minimum Microbicidal Concentration (MMC) of Colored Wheat Anthocyanin Extracts Against Human Pathogens

The results revealed the high and promising antimicrobial activity of colored wheat anthocyanin extracts against the human pathogens in comparison to white wheat extracts.

All the extracts showed dose-dependent antimicrobial activity and showed the highest activity at 200 mg/mL extract concentration. Maximum antimicrobial activity was shown by black wheat flour extracts, followed by purple flour extracts (Table 3). Black wheat flour showed MIC against all the pathogens in the range of 50–150 mg/mL, with the highest (50 mg/mL) against *S. aureus* and *P. aeruginosa*. The MMC of 100 and 150 mg/mL of black wheat flour extract completely inhibited the growth of *S. aureus* and *P. aeruginosa*, respectively. *E. coli* and *C. albicans* were inhibited only at the higher concentration of 200 mg/mL. Purple wheat flour (50–150 mg/mL) showed MIC against all pathogens and showed an MMC of 150 mg/mL against *S. aureus* and *P. aeruginosa*. However, among colored wheat flour extracts, blue wheat showed minimum antimicrobial activity. It showed MMC of 200 mg/mL against *S. aureus* and *P. aeruginosa* and MIC of 100 mg/mL against *P. aeruginosa* and 150 mg/mL against *S. aureus* and *E. coli*. It did not show any MIC against *C. albicans*. In comparison to colored wheat flours, white wheat flour extracts did not show any MMC against *E. coli* and *C. albicans*. It showed MIC of 100 mg/mL against *S. aureus*, *P. aeruginosa*, and *E. coli*, and 200 mg/mL against *C. albicans*, and did not show any antimicrobial activity below 100 mg/mL extract concentration.

Black wheat-grass juice extracts showed MIC against all the pathogens in the range of 100–150 mg/mL, with the highest activity against *P. aeruginosa* and *C. albicans*. However, black wheat-grass juice extracts showed MMC (200 mg/mL) against *S. aureus* and *P. aeruginosa* only. Purple wheat-grass juice extracts showed MMC of 200 mg/mL against *S. aureus* and MIC of 150 mg/mL against *S. aureus* and *P. aeruginosa*. Among all the colored wheat extracts, blue wheat-grass juice extracts showed minimum antimicrobial activity. Blue wheat-grass juice extracts did not show any MMC against any of the pathogens and showed MIC of 150 mg/mL against *P. aeruginosa* and 200 mg/mL against *S. aureus* and *E. coli*. It did not show any MIC against *E. coli* and *C. albicans*. Similarly, white wheat-grass juice extracts did not show MMC against any of the pathogens and showed MIC of 150 mg/mL against *S. aureus* and *P. aeruginosa* and 200 mg/mL against *E. coli*. Antibiotics in the concentration of (10 µg/mL) completely inhibited the growth of all the pathogens.

## 3. Discussion

Anthocyanins are widely recognized for their antioxidant potential and several health benefits, and anthocyanin-rich colored wheats are gaining the interest of consumers globally [27,28,29]. The results showed that TAC content in colored wheat flours and wheat-grass juices were significantly higher as compared to white wheat flour and white wheat-grass, respectively. These results are in accordance with Abdel-Aal et al. [30], Abdel-Aal and Hulc [31], Sharma et al. [28], and Liu et al. [32], who reported significantly higher TAC contents in colored wheat flours in comparison to white wheat flours. Similarly, Sytar et al. [17] found that sprouts of colored wheat grains had higher TAC content and antioxidant activity in comparison to white wheat sprouts. Anthocyanins are well known for sequestering free radicals generated during various metabolic processes inside the body. Free radicals cause oxidative damage to DNA and other biological molecules. In this study, DPPH and ABTS assays were used to analyze the free radical cation quenching potential of different colored wheat flours and wheat-grass juices. The extent of discoloration of ABTS and DPPH reagents is used to evaluate the percent inhibition of the ABTS or DPPH radicals by the antioxidants present in a sample. In both the assays, black wheat flour showed the highest antioxidant potential among flour samples and black wheat-grass showed the highest potential among wheat-grass samples, while white wheat flour and white wheat-grass juice showed the least antioxidant potential amongst all. Similar results were obtained by Li et al. [33], Pasqualone et al. [34], Sharma et al. [28], Li and Beta [35], and Kumari et al. [36], who reported higher antioxidant activity of colored wheat compared to white wheat.

In our study, the determination of anthocyanins through UPLC authenticated and provided an insight into the composition and characterization of anthocyanins in different colored wheat flours and wheat-grasses. UPLC analysis revealed the presence of six anthocyanins peaks in all colored wheat flour anthocyanin extracts. These were delphinidin-3-o-galactoside chloride, delphinidin-3-o-glucoside chloride, delphinidin-3-o-rutinoside chloride, cyanindin-3-o-glucoside chloride, pelargonidin 3-o-glucoside chloride, and peonidin-3-o-rutinoside chloride. All these anthocyanins have been reported previously in the case of colored cereals [19,26,37,38]. In our study, delphinidin-3-o-galactoside chloride, delphinidin-3-o-glucoside chloride, and cyanindin-3-o-glucoside chloride were found to be the major anthocyanins in black wheat flour extracts. Cyanidin and delphinidin have very high free radical scavenging potential and are reported to possess antiproliferative and apoptotic effects in MCF7 human breast cancer [39]. Studies have shown cyanidin 3-glucoside to be the major anthocyanin in black rice [40], and blue and purple corns [26]. Hao et al. [41] showed the presence of cyanidin 3-glucoside, cyanidin 3-rutinoside, and peonidin 3-glucoside in black rice. These anthocyanins are also present in our black wheat extracts. In our previous study, we had shown the presence of glucosides or rutinosides linked cyanidin, delphinidin, pelargonidin, petunidin, malvidin, and peonidin in black wheat [19]. However, in comparison to all other colored wheats, black wheat anthocyanins are comparatively less characterized.

In blue wheat flour extracts, major anthocyanins were malvidin-3-o-glucoside chloride, delphinidin-3-o-galactoside chloride, cyanindin-3-o-glucoside chloride, and petunidin-3-o-glucoside chloride. Many studies have found cyanidin 3-glucoside, cyanidin 3-rutinoside, delphinidin 3-glucoside, delphinidin 3-rutinoside, petunidin-3-glucoside, petunidin-3-rutinoside, and malvidin 3-glucoside to be the principal anthocyanins in blue wheat [26,27,42,43,44]. These anthocyanins are present in our blue wheat extracts as well. Other studies have shown the presence of cyanidin-3-glucoside and pelargonidin-3-glucoside in blue maize [45,46,47].

Purple wheat flour extracts had a different chromatographic pattern of anthocyanins in comparison to black and blue wheat extracts. The highest concentration of cyanindin-3-o-glucoside chloride followed by pelargonidin 3-o-glucoside chloride was observed in purple wheat flour extracts. One previous study has also shown cyanindin-3-o-glucoside chloride and pelargonidin 3-o-glucoside chloride as the major anthocyanins in purple wheat [48]. Our results are also in accordance with Hosseinian et al. [49], who showed the presence of delphinidin 3-galactoside, cyanidin 3-glucoside, and pelargonidin 3-glucoside in purple wheat.

Anthocyanins from colored wheat-grasses have been characterized much less in comparison to colored wheat flours. In the case of wheat-grass extracts, pelargonidin 3-o-glucoside chloride was present in all the extracts. Sytar et al. [17] had studied the anthocyanin composition in sprouts of blue, purple, and yellow wheat grains and found pelargonidin and cyanidin to be the major anthocyanins present in these grains. In our study, the major anthocyanins present in black wheat-grass juice extract were cyanindin-3-o-glucoside chloride and pelargonidin 3-o-glucoside chloride. Purple wheat-grass juice extracts also had the highest content of pelargonidin 3-o-glucoside chloride. It is reported to have antidiabetic and anti-inflammatory effects [50,51].

There has been growing interest nowadays in identifying natural therapeutic and antimicrobial agents. The antimicrobial activity of wheat flour and wheat-grass juice extracts has been previously reported, particularly against *S. aureus*, *E. coli*, *P. aeruginosa*, and *C. albicans* [12,52,53]. However, there are no reports on the antimicrobial properties of anthocyanin extracts from colored wheat. In the present study, the antimicrobial activity of anthocyanins extracted from black, purple, blue, and white wheat flour and wheat-grass juice extract was studied and tested against Gram-positive, Gram-negative, and yeast strains at different anthocyanin extract concentrations. The zone of inhibition obtained was dose-dependent and with the decrease in concentration, the zone of inhibition decreased subsequently.

Among flour extracts, black wheat flour showed maximum antimicrobial activity and was most effective in controlling the growth of all the microbial strains. All these pathogens are common human pathogens and colored wheat extracts proved to be a potent source for inhibiting their growth. Black and purple flour extracts were active against *S. aureus* and *P. aeruginosa*, even at a very low concentration of 50 mg/mL in comparison to white wheat. Purple wheat flour also exhibited high antagonism against the pathogens and showed MMC of 150 mg/mL and MIC of 50 mg/mL against *S. aureus* and *P. aeruginosa*. Among colored wheat flour extracts, blue wheat exhibited minimum antagonistic activity against these pathogens. White wheat flour, poor in anthocyanins, exhibited moderate activity, did not show any MMC against *E. coli* and *C. albicans*, and was effective against *P. aeruginosa* and *S. aureus* at higher concentrations (100 mg/mL and above) in comparison to colored wheat extracts. The inhibitory activity of the flour anthocyanin extracts was in the order: black > purple > blue > white. Colored wheat flour had higher antimicrobial activity against *E. coli*, *P. aeruginosa*, *C. albicans*, and *S. aureus* as compared to normal white wheat. Among colored wheat flours, black wheat was the most effective. This can be related to the higher anthocyanin content in black wheat flour and wheat-grass juice as compared to purple, blue, and white wheat. Higher anthocyanin content in black wheat and the presence of different anthocyanins, viz., pelargonidin 3-glucoside, cyanidin 3-glucoside, cyanidin 3-rutinoside, peonidin 3-galactoside, and cyanidin chloride through RP-UPLC and MS/MS analysis has been reported in our previous studies [19,28].

Similarly, among the wheat-grass juice extracts, black wheat-grass juice extract was most effective against the pathogens and showed MIC of 100–150 mg/mL against all the pathogens. It showed MMC of 200 mg/mL against *E. coli* and *C. albicans*; and 100 and 150 mg/mL against *S. aureus* and *P. aeruginosa.* In comparison to colored wheat-grass juice, white wheat-grass juice extracts did not show MMC against any of the pathogens. At a 200 mg/mL concentration, black and purple wheat-grass extracts showed a maximum zone of inhibition against *S. aureus*, *P. aeruginosa*, *E. coli*, and *C. albicans* as compared to white wheat, which did not show any activity against *C. albicans.* Wheat-grass has earlier been reported for having high antimicrobial activity [14,15]. It has shown high antimicrobial activity against *Streptococcus mutants* and *Lactobacillus* spp. [54]. Das et al. [55] reported activity in 80% acetone extracted wheat-grass samples against four bacteria—*B. cereus*, *S. aureus*, *E. coli*, and *Shigella flexneri*—and one fungus—*Aspergillus niger*. Wheat-grass significantly inhibited the growth of *E. coli* and can be a good alternative to chemical antibiotics. Deshwal and Deepshikha [56] observed the antimicrobial activity of 50% (*v/v*) anthocyanin extract in water and foundan inhibition zone of 28.66 mm against *E. coli*. However, all these results pertain to white wheat-grass. Wheat-grass has been reported to have high nutritional value and is a rich source of various vitamins, antioxidants, and minerals [3]. In our study, we have found white wheat-grass to be the least effective in comparison to colored wheat-grass, which has very high antagonistic activity against these pathogens even at a low concentration of 50 mg/mL. Besides showing strong antimicrobial activity, colored wheat is a potential nutraceutical agent and is reported to have high antioxidant potential and possess anti-inflammatory, antidiabetic, and antiobesity effects in mice-based models [28,29]. Hence, colored wheat consumption can lead to numerous health benefits mainly because of their high anthocyanin content and these have been associated with protection against different pathogens.

Many studies have reported that the antibacterial activities of plant extracts have been linked to the presence of some bioactive compounds that protect the plants themselves against bacterial, fungal, and viral infections [57,58]. Burdulis et al. [21] observed that extracts of berry and berry skin rich in anthocyanins showed inhibitory effects against Gram-positive (*L. monocytogenes*, *S. aureus*, *B. subtilis*, and *Enterococcus faecalis*) and Gram-negative bacterial strains (*Citrobacter freundii*, *E. coli*, *P. aeruginosa*, and *Salmonella enterica* ser. Typhimurium). Pomegranate fruit (*Punica granatum*) also contains a high proportion of phenolic compounds, and showed antimicrobial effect against *B. subtilis*, *Corynebacterium diphtheriae*, *S. aureus*, *S. epidermidis*, *S. saprophyticus*, *Enterococcus faecium*, *E. faecalis*, *Streptococcus pneumoniae*, and *E. coli* [59]. Carrot (*Daucus carota* L.) has high recognition and economic importance due to the presence of higher concentrations of bioactive compounds [60]. Acetone and ethanol extracts of black carrot exhibited antimicrobial activity against *S. aureus*, *B. cereus*, *E. coli*, and *Pseudomonas* spp. [61]. Not only do fruits or vegetables have high antimicrobial activity, cereals too have antimicrobial and antioxidant activity. According to a recent in vivo study, black rice extracts showed antibacterial and anti-inflammatory effects against the ulcer- and gastroduodenal diseases-causing bacteria *Helicobacter pylori* [62]. It has been reported that the seed coat extract of finger millet (*Eleusine coracana*) shows antimicrobial effect against *B. cereus* and *Aspergillus flavus* [63]. The mode of action of these extracts is via inhibition of DNA replication, protein synthesis, and breaking cell wall integrity [64].

## 4. Materials and Methods 

### 4.1. Plant Material

Plant material included three colored wheat varieties of *Triticum aestivum* viz., black wheat [65] (NABIMG-11), purple wheat [66] (NABIMG-10), blue wheat [67] (NABIMG-9), and one white wheat (PBW621). All the colored wheat varieties were developed at the National Agri-Food Biotechnology Institute (NABI), Mohali, Punjab, India through breeding techniques for anthocyanin biofortification using donor colored wheat lines and recipient white wheat variety (PBW 621) [28,39]. White wheat variety (PBW621) is a popular commercial high yielding wheat variety among Indian farmers. Wheat varieties were grown in the farms of NABI, Mohali, Punjab, India (30°44′10” N latitude at an elevation of 351 m above sea level) in 2018–2019 in late-October and harvested in mid-April.

### 4.2. Preparation of Wheat Flour

**Figure 5:**
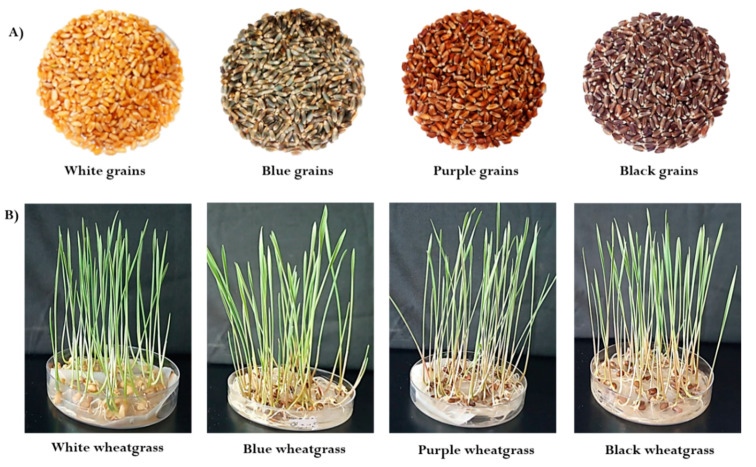
Different colored wheat grains (**A**); and wheat-grasses (**B**) used in the present study.

Four wheat varieties of different color—white, purple, blue, and black—were used in the present study (Figure 5A). Wheat grains were cleaned thoroughly to remove any dirt, dust, insect excreta, or other food grains and then, grounded in an electric grinder with 0.5 mm mesh sieves to make whole wheat flour. Flour samples were kept in airtight containers for future use.

### 4.3. Preparation of Lyophilized Colored Wheat-Grass Juice

For wheat-grass production, seeds were washed with distilled water, then sterilized with 4% sodium hypochlorite for 2 min, and put in sterilized Petri dishes with absorbent pads. Seeds were watered regularly, and germination proceeded under controlled conditions in a growth chamber with the following parameters: Relative humidity of 60–70% and a light/dark regime of 16/8 h at 25/20 °C (Figure 5B). Wheat-grass was harvested on the 10th day; wheat-grass juice was extracted by grinding fresh leaves in an electric mixer grinder and filtering the juice through a muslin cloth. Wheat-grass juice was lyophilized and stored at 4 °C for further anthocyanin content, antioxidant potential, and antimicrobial analyses.

### 4.4. Microbial Cultures

Microbial cultures of human pathogens were procured from the microbial type culture collection and gene bank facility (MTCC) of the Institute of Microbial Technology (IMTECH), Chandigarh. These included one Gram-positive bacteria, *S. aureus* (MTCC 1934); two Gram-negative—*P. aeruginosa* (MTCC 1434) and *E. coli*; and one yeast strain, *C. albicans* (MTCC 227). The bacterial cultures *S. aureus*, *P. aeruginosa*, and *E. coli* were grown on Trypticase soya agar (TSA) at 37 °C for 24–48 h, while *C. albicans* were grown on TSA and Sabouraud dextrose agar (SDA) at 28 °C for 24 and 72 h, respectively. All test microorganisms were maintained on TSA slants at 4 ± 0.2 °C. The microbial cultures were preserved in 25 percent (*v*/*v*) glycerol solution and stored at −80 °C for future use.

### 4.5. Total Anthocyanin Content (TAC) in Wheat Flour and Wheat-Grass

Anthocyanins were extracted using the method given by Boeing et al. [68]. Two grams of each colored wheat flour and lyophilized wheat-grass juice were taken and 20 mL of acidified methanol (85:15, *v*/*v*; methanol:1N HCl) was added. Samples were kept overnight at 28 °C in an incubator shaker with constant shaking. Thereafter, samples were centrifuged at 7000 rpm for 30 min at 4 °C, and supernatants were collected and filtered through a PVDF syringe filter (0.45 μm) [28]. Anthocyanin extraction was performed in triplicate. Total anthocyanin content (TAC) was determined using spectrophotometric methods. The absorbance of samples was measured at 520 nm, against distilled water as the blank. The data were expressed as micrograms (μg) of cyanidin 3-glucoside (Cy 3-glu) equivalents per gram of dry matter using the formula given by Young and Abdel-Aal [69]:

C = (A/e) × (V/1000) × MW × (1/sample wt) × 10^6^

where C is the concentration of total anthocyanin (mg/kg), A is absorbance of the sample, e is molar absorptivity of cyanidin 3-glucoside (25,965 cm^–1^ M^–1^), V is total volume of anthocyanin extract used, and MW is the molecular weight of cyanidin 3-glucoside.

For carrying out the antimicrobial studies, anthocyanins were concentrated in a rotavapor at 37 °C and dried extracts were dissolved in 10% sterile Dimethyl Sulphoxide (DMSO) at the final concentration of 200 mg/mL.

### 4.6. Anthocyanin Determination by Ultra Performance Liquid Chromatography (UPLC)

#### 4.6.1. Anthocyanin Standards

Sixteen different anthocyanin standards were procured from Extrasynthase manufacturers. These were cyanin chloride (cyanidin-3,5-di-o-glucoside chloride) [C], cyanindin-3-o-glucoside chloride [CG], cyanindin-3-o-rutinoside chloride [CR], delphin chloride (delphinidin-3,5-di-o-glucoside chloride) [DCH], delphinidin chloride (3,3′,4′,5,5′,7-hexahydroxyflavylium chloride) [D], delphinidin-3-o-glucoside chloride [DG], delphinidin-3-o-rutinoside chloride [DR], delphinidin-3-o-sambubioside chloride [DS], delphinidin-3-o-galactoside chloride [DGL], malvin chloride (malvidin-3,5-di-o-glucoside chloride) [MC], malvidin-3-o-glucoside chloride [MG], peonidin-3-o-glucoside chloride [POG], peonidin-3,5-di-o-glucoside chloride [PODG], peonidin-3-o-rutinoside chloride [POR], petunidin-3-o-glucoside chloride [PTG], and pelargonidin 3-o-glucoside chloride [PLG]. Stock solutions of 1 mg/mL were prepared for all the standards and standard curves were made in the concentrations ranging from 1 to 200 ppm.

#### 4.6.2. UPLC Analysis

For UPLC analysis, the anthocyanin extracts of wheat flour and wheat-grass juice were prepared as explained in Section 4.5. The extracts were concentrated in a rotavapor at 37 °C and dried extracts were dissolved in 1 mL methanol. UPLC of these anthocyanin extracts was performed using Waters Acquity UltraPerformance™ LC system (Waters corporation, Milford, The United States of America), equipped with a quaternary pump system according to the method given by Sharma et al. [28], with slight modifications. An Acquity BEH C-18 (50 × 2.1 mm id, 1.7 μm particle size) column (Waters) was used for analysis. Detection was carried out using a photodiode array (PDA) detector with the absorbance wavelength of 520 nm. The injection volume was 2 μL, column temperature was set at 50 °C, and the flow rate was 0.5 mL min^−1^. Eluent A comprised 5% (*v*/*v*) formic acid and eluent B comprised HPLC grade acetonitrile. The gradient run was 0–5.0 min: 95% A and 5% B; 5.0–5.1 min: 85% A and 15% B (curve 6); 5.1–6.0 min: 84.5% A and 15.5% B (curve 6); 6.0–6.1 min: 84.5% A and 15.5% B (curve 6); 6.1–6.8 min: 0% A and 100% B (curve 6). The separation was carried out for 6.8 min in the gradient elution. The identification of anthocyanins present in wheat flour and wheat-grass juice extracts was performed by comparing the retention times of the anthocyanin peaks in sample extracts with the peaks in standard solutions. Anthocyanin concentrations in wheat samples were calculated from calibration curves. Three replicates of each sample were used for detection and quantification of anthocyanins.

### 4.7. Antioxidant Potential of Colored Wheat Flour and Wheat-Grass Juice

The antioxidant potential of wheat flour and wheat-grass was studied using two methods:

#### 4.7.1. DPPH Radical Scavenging Assay

The antioxidant activity of the methanol extracts of anthocyanins from colored wheat flour and lyophilized wheat-grass juice was measured using 1, 1-diphenyl-2-picrylhydrazyl (DPPH) assay [70], in which the potential of the test samples to scavenge DPPH is tested. In total, 100 µL of methanol extracts of anthocyanins from wheat flour and wheat-grass juice samples (prepared in Section 4.5) were added to 3.9 mL freshly prepared methanolic DPPH (60 µmol/L) solution. Mixtures were incubated for 30 min in the dark at room temperature and thereafter, absorbance was recorded at 517 nm. Methanol was taken as the blank and 100 µL methanol and 3.9 mL DPPH solution was taken as the control. The antioxidant activity was calculated in terms of percentage (%) inhibition of DPPH using the formula:

Percentage inhibition (%) = [(A_control_ − A_sample_)/A_contro_l)] × 100 (1)

where A_control_ is the absorbance of the control reaction (containing all reagents except test compound); and A_sample_ is the absorbance of the test compound. All tests were carried out in triplicate.

#### 4.7.2. ABTS Radical Scavenging Assay

The antioxidant activity of the methanol extracts of anthocyanins from colored wheat flour and wheat-grass juice was also measured using the ABTS assay [70]. One ml of ABTS reagent was prepared by mixing equal volumes of ABTS stock solution (7 mM) and potassium persulfate stock solution (2.6 mM) in methanol and incubating for 16 h in the dark at room temperature. Thereafter, the absorbance of the reagent was adjusted to 0.7 ± 0.02 at 734 nm by diluting the solution further with methanol. A total of 100 µL of methanol extracts of anthocyanins from wheat flour and wheat-grass juice samples (prepared in Section 4.5) were added to 3.9 mL of freshly prepared ABTS solution. After 1h of incubation at room temperature, absorbance of the solutions was recorded at 734 nm. The blank consisted of methanol and the control consisted of 100 µL methanol and 3.9 mL ABTS solution. The antioxidant activity was calculated in terms of percentage (%) inhibition of ABTS, using the formula:

Percentage inhibition (%) = [(A_control_ − A_sample_)/A_contro_l)] × 100 (2)

where A_control_ is the absorbance of the control reaction (containing all reagents except test compound); and A_sample_ is the absorbance of the test compound. All tests were carried out in triplicate.

### 4.8. Antimicrobial Activity of Colored Wheat Anthocyanins Against Microbial Strains Using Agar-Overlay Method

The antagonistic activity of anthocyanins extracted from wheat flour and wheat-grass juice towards human pathogens was tested using the agar overlay method, as described by [71]. The microbial strains were subcultured by inoculating a single colony of the strain in the 10 mL of Trypticase soya broth (TSB) and incubated for 24–48 h to obtain the viable count of 10^7^ cells/mL. Sabouraud dextrose broth (SDB) was used in the case of *C. albicans.* To prepare a homogeneous lawn of bacteria, 100 µL of 24–48 h old microbial cultures were mixed in 100 mL of soft TSA (0.7% agar) and poured over the thin layer of TSA in Petri plates. Soft agar was used because extracts diffuse properly in it as compared to normal agar. Similarly, SDA was used in the case of *C. albicans.* After solidification, 4 wells of 5 mm diameter were made in each plate using a sterile cork borer, and filled with 100 µL of different concentrations of anthocyanins extracted from wheat and wheat-grass juice (50, 100, 150, 200 mg/mL DMSO). The negative control consisted of 100 µL of uninoculated TSB and the positive control was 100 µL of antibiotic streptomycin (10 µg/mL). Incubation time varied from 24–48 h at 28 ± 2 °C for *C. albicans* to 24 h at 37 °C for *S. aureus*, *P. aeruginosa*, and *E. coli*. The size of the inhibition zone around the wells was recorded and the antimicrobial activity (mm) was expressed as the difference between diameter of the inhibition zone around the well and diameter of the well [72].

### 4.9. MIC and MMC of Colored Wheat Anthocyanin Extracts Against Human Pathogens

The broth microdilution method was used to determine the MIC and MMC of colored wheat in 96-well plates [73]. Stock solutions were prepared by dissolving test extracts in 10% sterilized DMSO. Twofold dilution series of test extracts were prepared for the dose range 10–200 mg/mL in sterilized TSB in 96-well microtiter plates. SDB was used for preparing serial dilution of *C. albicans*. The plates were inoculated with freshly grown bacterial cultures adjusted to a concentration of 10^7^ cfu/mL with sterile normal saline. Briefly, 100 µL of 2x TSB was poured in each well and 10 µL aliquot of the serial dilution starting from lower dilution in the first well (0, 25, 50, 75, 100, 150, 200 mg/mL DMSO) prepared from the test sample was added to the wells in each row. Each well in a row having serial dilutions was inoculated with 10 µL of the test microorganism and the final volume in each well was made up to 200 µL with sterilized distilled water. The uninoculated sterilized medium with and without DMSO served as the control. The antibiotic streptomycin in the concentration of 10 µg/mL served as the positive control. The inoculated control consisted of 10 µL of the test microorganism without wheat anthocyanin extracts.

The plates were kept for 24–48 h at 28 ± 2 °C for *C. albicans,* and for 24 h at 37 °C for *S. aureus*, *P. aeruginosa*, and *E. coli*. After incubation time, MIC was recorded as the lowest extract concentration which inhibited the visible microbial growth when compared to the inoculated control. MMC was determined by spread plating the 100 µL aliquot from dilutions and assessing growth on TSA medium for bacterial cultures and SDA for *C. albicans*. The lowest concentration which completely killed the inoculated microorganisms was recorded as the MMC.

### 4.10. Statistical Analysis

All experiments were performed in triplicate and the results were expressed as means ± standard deviation (SD). Results were analyzed using one-way ANOVA and Tukey test (5% probability) using the IBM SPSS 21.0 software (IBM, Armonk, New York, NY, USA) and Graph Pad Prism 7 (GraphPad Software, San Diego, CA, USA).

## 5. Conclusions

The study shows that anthocyanin-rich colored wheat flour and wheat-grass juice possess strong antioxidant capacity against free radicals and have high antimicrobial potential against various human pathogens in comparison to white wheat. Hence, colored wheat flour and wheat-grass juice are natural plant-based antimicrobial agents which are capable of providing health benefits to the consumers.

## Figures and Tables

**Table 1:** Concentration of different anthocyanins (ppm) present in wheat flour and wheat-grass juice extracts.

	Wheat Flour				Wheat-Grass			
Anthocyanins	Black	Purple	Blue	White	Black	Purple	Blue	White
DGl	29.14 ± 0.87 ^g^	0.40 ± 0.02 ^b^	4.95 ± 0.04 ^f^	0.03 ± 0.01 ^a^	-	-	-	-
DG	25.64 ± 0.69 ^f^	0.08 ± 0.00 ^a^	0.40 ± 0.01 ^a^	-	-	-	-	-
DR	0.66 ± 0.06 ^ab^	-	2.08 ± 0.09 ^c^	-	2.03 ± 0.08 ^b^	0.21 ± 0.03 ^b^	2.36 ± 0.05 ^d^	-
CG	20.50 ± 1.06 ^e^	2.64 ± 0.04 ^e^	4.50 ± 0.03 ^e^	0.07 ± 0.01 ^b^	4.74 ± 0.51 ^d^	0.23 ± 0.02 ^b^	-	0.37 ± 0.04 ^b^
PODG	0.23 ± 0.07 ^a^	-	0.31 ± 0.10 ^a^	-	-	-	-	0.26 ± 0.04 ^a^
PTG	2.29 ± 0.21 ^c^	-	3.18 ± 0.30 ^d^	-	-	-	-	-
PLG	2.13 ± 0.05 ^c^	1.88 ± 0.06 ^d^	0.39 ± 0.09 ^a^	-	2.77 ± 0.11 ^c^	0.41 ± 0.03 ^d^	0.40 ± 0.03 ^c^	0.35 ± 0.02 ^b^
POG	1.40 ± 0.09 ^bc^	-	-	-	1.03 ± 0.15 ^a^	0.32 ± 0.06 ^c^	0.14 ± 0.01 ^a^	-
POR	0.97 ± 0.10 ^ab^	-	0.76 ± 0.26 ^b^	-	-	0.18 ± 0.03 ^ab^	0.31 ± 0.03 ^b^	-
MG	2.18 ± 0.17 ^c^	1.32 ± 0.04 ^c^	5.50 ± 0.15 ^g^	0.12 ± 0.01 ^c^	1.25 ± 0.17 ^a^	-	0.15 ± 0.01 ^a^	-
CR	11.14 ± 0.25 ^d^	0.20 ± 0.01 ^ab^	0.60 ± 0.17 ^ab^	-	-	-	-	-
DCH	-	-	-	-	1.89 ± 0.30 ^b^	0.14 ± 0.01 ^a^	-	-

Values are the means of three replicates ± standard deviation. Different letters in each column represent significantly different values (*p* < 0.05) designated as a < b < c. Similar letters in the column represent values being statistically on par. DGl—delphinidin-3-o-galactoside chloride; DG—delphinidin-3-o-glucoside chloride; DR—delphinidin-3-o-rutinoside chloride; CG—cyanindin-3-o-glucoside chloride; PODG—peonidin-3,5-di-o-glucoside chloride; PTG—petunidin-3-o-glucoside chloride; PLG—pelargonidin 3-o-glucoside chloride; POG—peonidin-3-o-glucoside chloride; POR—peonidin-3-o-rutinoside chloride; MG—malvidin-3-o-glucoside chloride; CR—cyanindin-3-o-rutinoside chloride; DCH—delphin chloride (delphinidin-3,5-di-o-glucoside chloride).

**Table 2:** Zones of inhibition (cm) in the growth of human pathogens by anthocyanin extracts from colored wheat flour and wheat-grass juice.

Wheat Sample	Extract Conc.	*S. aureus*		*P. aeruginosa*		*E. coli*		*C. albicans*	
	(mg/mL)	WF ^1^	WG ^2^	WF ^1^	WG ^2^	WF ^1^	WG ^2^	WF ^1^	WG ^2^
Black	200	2.50 ± 0.05 ^k^	2.23 ± 0.06 ^k^	2.73 ± 0.04 ^g^	2.60 ± 0.10 ^i^	2.50 ± 0 ^h^	1.93 ± 0.04 ^g^	2.57 ± 0.04 ^g^	2.37 ± 0.04 ^i^
	150	1.87 ± 0.06 ^g^	1.77 ± 0.04 ^i^	2.37 ± 0.06 ^def^	197 ± 0.12 ^g^	2.07 ± 0.05 ^f^	1.60 ± 0.02 ^ef^	2.03 ± 0.06 ^e^	2.03 ± 0.06 ^h^
	100	1.63 ± 0.05 ^f^	1.27 ± 0.05 ^fg^	2.03 ± 0.12 ^c^	1.46 ± 0.08 ^f^	1.53 ± 0.06 ^c^	1.03 ± 0.05 ^d^	1.83 ± 0.03 ^d^	1.77 ± 0.03 ^g^
	50	1.23 ± 0.04 ^c^	0.83 ± 0.06 ^d^	1.63 ± 0.12 ^b^	1.17 ± 0.04 ^e^	1.13 ± 0.06 ^ab^	0.77 ± 0.12 ^bc^	1.17 ± 0.04 ^b^	1.17 ± 0.04 ^de^
Purple	200	2.27 ± 0.06 ^j^	2.20 ± 0.10 ^k^	2.60 ± 0.10 ^fg^	2.27 ± 0.07 ^h^	2.37 ± 0.04 ^gh^	2.00 ± 0.10 ^g^	2.40 ± 0.10 ^f^	1.93 ± 0.12 ^gh^
	150	2.07 ± 0.05 ^hi^	1.87 ± 0.06 ^ij^	2.13 ± 0.12 ^cd^	1.87 ± 0.05 ^g^	1.97 ± 0.12 ^e^	1.80 ± 0.07 ^fg^	2.03 ± 0.12 ^e^	1.50 ± 0.03 ^f^
	100	1.77 ± 0.07 ^fg^	1.07 ± 0.05 ^e^	2.07 ± 0.05 ^c^	1.03 ± 0.05 ^de^	1.80 ± 0.10 ^de^	1.40 ± 0.10 ^e^	1.67 ± 0.06 ^c^	1.00 ± 0.10 ^d^
	50	1.43 ± 0.12 ^de^	0.57 ± 0.03 ^c^	1.57 ± 0.11 ^b^	0.50 ± 0 ^b^	1.20 ± 0.10 ^ab^	0.90 ± 0.04 ^cd^	1.57 ± 0.06 ^c^	0.57 ± 0.08 ^c^
Blue	200	1.93 ± 0.06 ^gh^	1.53 ± 0.05 ^h^	2.47 ± 0.05 ^ef^	1.53 ± 0.04 ^f^	2.30 ± 0.10 ^g^	1.10 ± 0.11 ^d^	1.53 ± 0.11 ^c^	1.53 ± 0.06 ^f^
	150	1.60 ± 0.10 ^ef^	1.17 ± 0.06 ^ef^	2.13 ± 0.05 ^cd^	0.97 ± 0.06 ^d^	2.07 ± 0.02 ^f^	0.87 ± 0.04 ^cd^	1.07 ± 0.05 ^b^	1.20 ± 0.10 ^e^
	100	1.30 ± 0.10 ^cd^	0.73 ± 0.02 ^d^	1.73 ± 0.12 ^b^	0.33 ± 0.06 ^b^	1.27 ± 0.03 ^b^	0.0 ± 0.0 ^a^	0.0 ± 0.0 ^a^	0.47 ± 0.05 ^c^
	50	0.0 ± 0.0 ^a^	0.0 ± 0.0 ^a^	0.97 ± 0.06 ^a^	0.0 ± 0.0 ^a^	1.07 ± 0.06 ^a^	0.0 ± 0.0 ^a^	0.0 ± 0.0 ^a^	0.0 ± 0.0 ^a^
White	200	2.13 ± 0.12 ^ij^	1.93 ± 0.06 ^j^	2.23 ± 0.07 ^cde^	2.17 ± 0.05 ^h^	2.47 ± 0.07 ^gh^	1.97 ± 0.12 ^g^	2.37 ± 0.07 ^f^	1.07 ± 0.15 ^de^
	150	1.77 ± 0.06 ^fg^	1.37 ± 0.04 ^g^	2.07 ± 0.12 ^c^	1.83 ± 0.05 ^g^	1.97 ± 0.11 ^ef^	1.43 ± 0.05 ^e^	2.03 ± 0.05 ^e^	0.20 ± 0.0 ^b^
	100	1.30 ± 0.10 ^cd^	0.87 ± 1.0 ^d^	1.63 ± 0.12 ^b^	1.37 ± 0.12 ^f^	1.67 ± 0.08 ^cd^	0.90 ± 0.09 ^d^	1.0 ± 0.10 ^b^	0.0 ± 0.0 ^a^
	50	0.83 ± 0.07 ^b^	0.27 ± 0.07 ^b^	0.97 ± 0.15 ^a^	0.77 ± 0.12 ^c^	1.53 ± 0.05 ^c^	0.57 ± 0.05 ^b^	0.0 ± 0.0 ^a^	0.0 ± 0.0 ^a^

1—WF (wheat flour); 2—WG (wheat-grass juice). Values are the means of three replicates ± standard deviation. Values followed by same letter in column are not significantly different (Tukey’s test, *p* > 0.05).

**Table 3:** Minimum inhibitory concentration (MIC) and minimum microbicidal concentration (MMC) of colored wheat anthocyanin extracts in mg/mL against human pathogens.

Wheat Sample	Inhibition	*S. aureus*		*P. aeruginosa*		*E. coli*		*C. albicans*	
		WF ^1^	WG ^2^	WF ^1^	WG ^2^	WF ^1^	WG ^2^	WF ^1^	WG ^2^
Black	MIC *	50	100	50	150	100	100	100	150
	MMC **	100	200	150	200	200	-	200	-
Purple	MIC *	50	150	50	150	100	150	150	150
	MMC **	150	200	150	-	-	-	-	-
Blue	MIC *	150	200	100	150	150	200	-	-
	MMC **	200	-	200	-	-	-	-	-
White	MIC *	100	150	100	150	-	200	200	-
	MMC **	200	-	150		-	-	-	-

* MIC—minimum inhibitory concentration; ** MMC—minimum microbicidal concentration; 1—WF (wheat flour); 2—WG (wheat-grass juice).
